# Fatigue and pain in children with multiple osteochondromas: a cross-sectional study

**DOI:** 10.1186/s13023-026-04476-2

**Published:** 2026-06-29

**Authors:** Ihsane Amajjar, Nienke W. Willigenburg, Rob J.E.M. Smeets, S. John Ham

**Affiliations:** 1 Department of Rehabilitation Medicine, CAPHRI, Maastricht University, Maastricht, The Netherlands; 2 Department of Orthopaedic Surgery, OLVG Hospital, Amsterdam, The Netherlands; 3 Pain in Motion International Research Group, www.paininmotion.be, Brussels, Belgium; 4 Clinics in Revalidatie (CIR), Eindhoven, The Netherlands

**Keywords:** Fatigue, Pain, Children, Multiple osteochondromas, Multiple variable regression analysis, Survey study

## Abstract

**Background:**

Multiple osteochondromas (MO) is a rare, inherited disorder characterized by multiple benign bone tumors. Although pain and fatigue are commonly encountered in clinical practice, their impact on children with MO remains largely unaddressed in literature. This study aimed to (1) assess fatigue and pain levels in children with MO, (2) compare fatigue severity with healthy peers, and (3) identify variables associated with fatigue and pain.

**Methods:**

In this cross-sectional study, 230 children (4-18yrs.) with MO were invited to complete validated, age-specific online questionnaires addressing fatigue (Checklist Individual Strength [CIS], visual analogue scale [VAS-fatigue]), pain (VAS), and psychosocial factors. Fatigue scores were compared to reference data from healthy peers using one-sample t-tests. The International Classification of Functioning, disability and health framework was used for a-priori selection of potential independent variables for multivariable regression analysis.

**Results:**

A total of 134 children participated. Fatigue was reported by 89.6% (VAS-fatigue = 3.17 ± 2.63), with CIS scores significantly higher than healthy controls (CIS-MO = 66.32 ± 24.63 vs. CIS-healthy peers = 55.07 ± 20.99, *p* = 0.012). Pain was reported by 75.4% (VAS = 2.41 ± 2.42). For fatigue, both CIS and VAS-fatigue were associated with pain and emotional/behavioral problems (CIS-model adj. R2 = 0.524, *p* < 0.001; VAS-fatigue model adj. R2 = 0.576, *p* < 0.001). CIS was also associated with depressive symptoms (CDI, *p* = 0.019). The pain regression model explained 45% of the variance (*p* < 0.001), and pain was significantly associated with functional disability and VAS-fatigue (*p* < 0.001).

**Conclusion:**

Fatigue, alongside pain, is highly prevalent and significantly increased in children with MO compared to healthy peers. Psychosocial factors are closely associated with both fatigue and pain, underscoring the importance of multidisciplinary disease management.

**Clinical trial number:**

Not applicable.

**Supplementary Information:**

The online version contains supplementary material available at https://doi.org/10.1186/s13023-026-04476-2.

## Introduction

Multiple osteochondromas (MO) is a chronic skeletal disorder that typically presents in early childhood and persists throughout life, resulting in substantial orthopedic morbidity. MO is characterized by the development of multiple benign bone tumors, osteochondromas, which may lead to a range of complications, including compression of nerves, vessels or tendons, restricted joint mobility, and functional limitations arising from skeletal deformities and growth disturbances. Among these clinical manifestations, pain has long been recognized as a prevalent and impactful symptom [1–4]. More recently, fatigue has emerged as an important but under-recognized contributor to disease burden in adults with MO [4, 5]. 

Fatigue was reported by 90.4% of the 353 adult patients with MO in a recent study by our team [4]. Bathen et al. reported higher fatigue scores than healthy controls in a cohort of 11 children with MO [5]. To our knowledge, these are the only studies that have specifically examined fatigue in MO, underscoring the limited research even more so among children.

Pain has been reported as highly prevalent in MO in the limited number of studies on this topic [1–4]. Some of these studies have sought to identify potential associations with other factors. Goud et al. documented pain in 63% of children with MO, a finding associated with school problems [3]. Darilek et al. found pain in 39.1% of participants under 20 years old, with a significant association of pain with both MO-related complications and surgical intervention [2]. 

Currently, no cure exists for MO, and treatment is limited to symptom management. This often involves conservative (i.e. non-surgical) therapy, but also various surgical interventions such as resection of symptomatic osteochondromas or limb reconstruction procedures. Interestingly, several studies have suggested surgery may paradoxically increase pain, advocating for a more conservative management strategy [1, 2, 6, 7]. Whether surgery initiates a pain cycle or whether widespread pain is already established, potentially through a nociplastic mechanism, remains unclear.

A potentially relevant variable of interest in MO research and treatment is gender, as its role in disease severity remains debated. Some studies suggest that males with MO are more severely affected, potentially due to a higher prevalence of EXT1 mutations and a longer period until skeletal maturity [8–12]. However, these findings are inconsistent, and it remains unclear whether gender is associated with fatigue and pain in MO.

Although the majority of the limited research on fatigue and pain in MO focuses on adults, children are not simply small adults, and the literature on these symptoms in pediatric patients with MO is notably lacking. This knowledge gap is particularly significant given that in other chronic pediatric diseases, fatigue and pain have been shown to negatively affect school performance, social development, and overall quality of life [13–16]. This potential burden underscores the need to clarify the associated variables that may influence fatigue and pain, which could inform future treatment strategies for MO. Extrapolation from other pediatric chronic disease populations may be inappropriate, as it is unknown whether these relationships are disease-specific or transdiagnostic. Therefore, the aims of this study are: (1) to assess the level of fatigue and pain in girls and boys with MO; (2) to compare the prevalence and severity of fatigue in MO to gender- and age-matched healthy subjects and (3) to identify associated variables for fatigue and pain in the pediatric MO population.

## Methods

### Study design and participants

This cross-sectional survey study was conducted from June 2019 to January 2022 in the Netherlands at the national MO expertise centre (OLVG Hospital, Amsterdam). Children aged 4 to < 18 years and diagnosed with MO were invited to participate via the hospital database and via the Dutch patient association ‘HME-MO Vereniging Nederland’. Non-Dutch speakers were excluded. Participants completed age-appropriate validated questionnaires during a routine outpatient visit or at home. A parent-proxy questionnaire was employed for children aged 4–7 years, while self-report versions were utilized for children aged 8–15 years and adolescents aged 16–17 years. Participation was voluntary, with withdrawal permitted at any time without explanation.

### Data collection

**Fig. 1:**
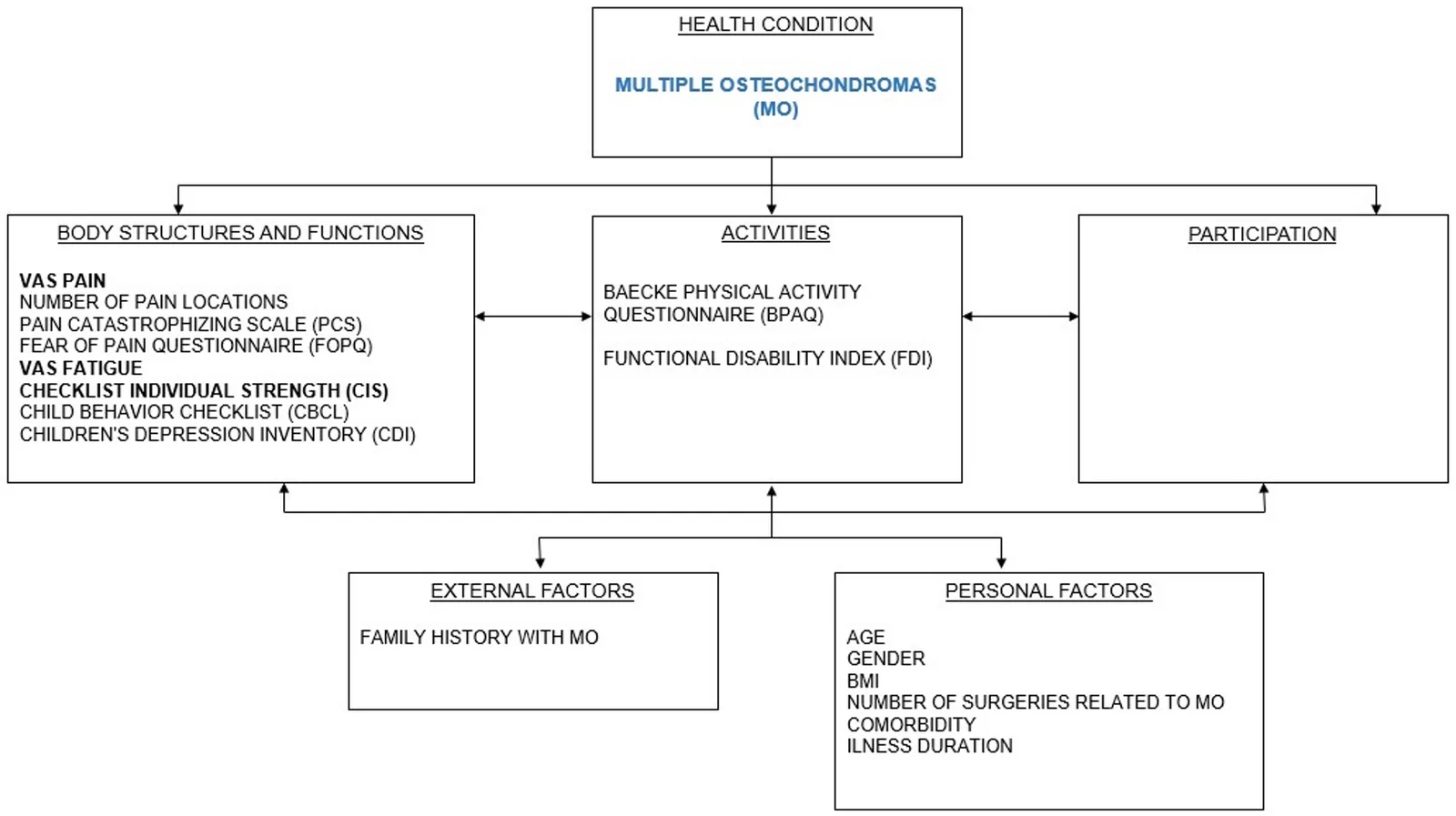
ICF model for fatigue and pain and possible associated independent variables.* In bold the measures for the dependent variables; fatigue and pain

Data was collected using the data capture system Castor EDC [17], which sent secure survey invitations via email and reduced the risk of missing data by preventing participants from skipping questions. Questionnaire selection was guided by a theoretical framework using the International Classification of Functioning, Disability and Health (ICF) model (Fig. 1), developed by the World Health Organization [18]. The primary outcomes, fatigue and pain, were assessed using validated questionnaires described below. Additional parameters, considered potentially associated independent variables, were selected in advance based on hypotheses formulated from a review of the literature and the ICF model. The hypotheses were: (1) children with MO experience more fatigue and pain than heathy peers; (2) pain intensity is independently associated with fatigue in children with MO; (3) fatigue and pain are associated with psychosocial variables and physical activity; (4) male gender, older age and a higher BMI are positively associated with fatigue and pain; (5) a greater number of pain sites, more surgical procedures, negative family history, and the presence of comorbidities are positively associated with fatigue and pain.

### Primary outcome measures

Both fatigue and pain were assessed using the Visual Analogue Scale (VAS), which asked participants to rate their average fatigue and pain intensity over the past week (range 0–10, where 0 indicates no fatigue/pain and 10 the worst imaginable fatigue/pain). Fatigue was also assessed with the validated Checklist Individual Strength questionnaire (CIS-total; range 20–140). Problematic fatigue is defined as a CIS-total score of > 76. The CIS-total comprises four domains of fatigue: (1) fatigue severity; (2) concentration problems; (3) motivation; (4) physical activity level [19]. Severe fatigue is defined as a score of ≥ 35 on the CIS-Subjective Fatigue subscale (range 8–56). Reference scores of Dutch healthy peers were used [20]. 

### Independent variables (potentially associated with fatigue and pain)

- Sociodemographic- and disease related factors were collected as listed in Table 1.
- The Pain Catastrophizing Scale (PCS; range 0–52) was used to assess the extent of pain catastrophizing, with higher scores reflecting greater levels of catastrophic thinking [21].
- The Fear of Pain Questionnaire (FOPQ; range 0–96) was used to evaluate children’s fear of pain and avoidance of activities because of this fear, with higher scores denoting greater fear [22, 23].
- The Functional Disability Inventory (FDI; range 0–60), was used to assess pain-related functional limitations in school, at home, during leisure activities, and in social interactions. Score interpretation: minimal disability (0 − 12), moderate disability (13–29), and severe disability (≥ 30) [24].
- The Baecke Physical Activity Questionnaire (BPAQ; range 3–15) was used to measure physical activity levels, with higher scores indicating higher levels of physical activity [25, 26].
- The Child Behavior Checklist (CBCL) includes both parent-proxy and self-report forms that assess a range of emotional and behavioral symptoms. For this study, total scores as well as the internalizing and externalizing subscale scores were analysed [27].
- The Children’s Depression Inventory (CDI; range 0–54) was used to assess depression, with higher scores indicating higher levels of depressive symptoms [28, 29].

The questionnaires employed in this study were validated and tailored to specific age groups, which explains the absence of certain data in the youngest participants. A detailed overview of the questionnaires and their applicability by age group can be found in Appendix A.

### Statistical analysis

All statistical analyses were performed using SPSS version 27 (IBM, Chicago, IL, USA). Descriptive statistics were used to report patient characteristics and prevalence rates of pain, fatigue, physical activity, and psychosocial variables. Gender differences were analysed using Chi-square tests or independent samples t-tests as appropriate. Fatigue scores (CIS) were compared to reference values from healthy peers using a one-sample t-test.

To examine associations between hypothesized independent variables and the outcomes fatigue and pain, a two-step purposeful selection method [30] was applied to construct three linear regression models with (1) VAS fatigue; (2) CIS total; and (3) VAS pain scores as dependent variables. Independent variables, selected a priori based on the ICF framework, were first analysed using univariable regression; those independent variables with a P value ≤ 0.25 were subsequently included in multivariable models. Backward selection was employed with a significance threshold of *p* ≤ 0.05 [30]. Final models were evaluated for multicollinearity (Variance Inflation Factor, VIF < 4, Tolerance > 0.1), normality of residuals, and homoscedasticity. No formal power analysis was conducted due to the rarity of MO and the limited number of eligible patients. Based on a previous study conducted by Goud et al. [3], which included 99 children with MO, a minimum sample size of 100 participants was targeted.

## Results

### Patient characteristics

**Fig. 2:**
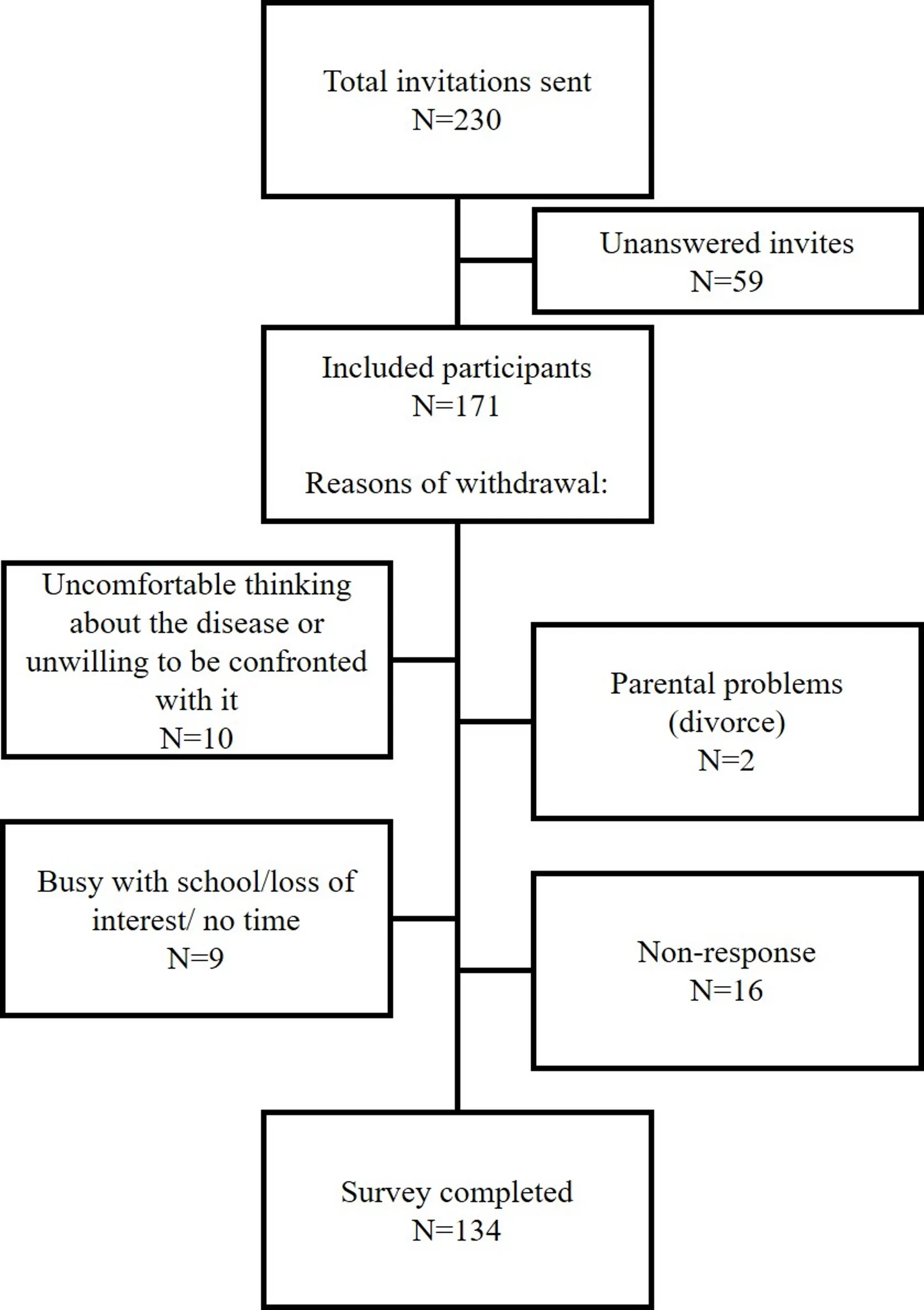
Included participants and outline of withdrawal reasons

Of the 230 invited children, 134 participants completed the survey, resulting in a response rate of 58%. The reasons for participant withdrawal are outlined in Fig. 2. Of the 134 participants, 47% were female, and the mean age of the total population was 11.4 ± 3.7 years old. Table 2 summarizes additional patient characteristics, most of which were similar between boys and girls. Female participants reported a significantly higher functional disability score compared with their male counterparts (mean difference = 3.78, *p* = 0.027, 95%CI -7.11; -0.45). A total of 12.6% of participants had a CBCL Total Problems score in the clinical or borderline range, indicating a substantial burden of behavioral and emotional problems among these children and adolescents with MO. CDI scores indicated that 7.7% of participants met criteria for clinically relevant depressive symptoms (CDI > 19), while 9.7% reported experiencing thoughts of suicide.

**Table 1:** Patient characteristics, clinical data and independent variables

	*N*	Total population Mean ± SD	Female Mean ± SD	Male Mean ± SD
**Age (years)**	134 (F:63 M:71)	11.4 ± 3.73	11.6 ± 3.48	11.2 ± 3.95
**BMI (kg/m** ^**2**^ **)**	134	18.2 ± 3.16	17.7 ± 2.96	18.7 ± 3.29
**Illness duration (years)**	134	7.53 ± 4.31	7.2 ± 4.34	7.8 ± 4.30
**Positive Family History MO**,** N (%)**	134	95 (70.9%)	46 (73%)	49 (69%)
**Comorbidities**,** N (%)**	134	15 (11.2%)	8 (12.7%)	7 (9.9%)
**Deformities of the extremities**,** N (%)**	134	99 (73.9%)	55 (87%)	44 (62%)
**Number of surgical procedures**	134	2.36 ± 3.25	2.4 ± 3.24	2.3 ± 3.27
**Number of pain location sites**	134	3.04 ± 3.09	3.5 ± 3.50	2.6 ± 2.63
**Pain medication usage (%)**	134	4 (3%)	3 (5%)	1 (1.4%)
**Pain Catastrophizing Scale**	134	10.61 ± 9.15	9.98 ± 9.3	11.17 ± 9.03
**Baecke Questionnaire**	76 (F:38 M:38)^a^	7.35 ± 1.01	7.31 ± 1.05	7.39 ± 0.98
**Child Behavior Checklist**	127 (F:63 M:64)^a^			
** Total problems**		30.25 ± 18.36	30.59 ± 18.98	29.92 ± 17.86
Normal range		111 (87.4%)	56 (89%)	55 (85.9%)
Borderline		6 (4.7%)	4 (6.4%)	2 (3.1%)
Clinical range		10 (7.9%)	3 (5.8%)	7 (10.9%)
** Internalizing problems**		11.0 ± 9.12	11.91 ± 9.75	10.11 ± 8.43
Normal range		91 (71.7%)	46 (73%)	45 (70.3%)
Borderline		11 (8.7%)	7 (11.1%)	4 (6.3%)
Clinical range		25 (19.7%)	10 (15.9%)	15 (23.4%)
**Externalizing problems**		9.69 ± 6.66	9.29 ± 6.33	10.09 ± 7.0
Normal range		103 (81.1%)	53 (84.1%)	50 (78.1%)
Borderline		14 (11%)	8 (12.7%)	6 (9.4%)
Clinical range		10 (7.9%)	2 (3.2%)	8 (12.5%)
Functional Disability Inventory*	104 (F:50 M:54)^a^	7.56 ± 8.57	9.52 ± 10.06*	5.74 ± 6.50*
**Children’s Depression Inventory**	104^a^	6.85 ± 6.66	7.92 ± 7.97	5.85 ± 5.06
**Fear of Pain Questionnaire**	76 (F:38 M:38)^a^	26.80 ± 17.31	26.66 ± 18.86	26.95 ± 15.86

* *p* < 0.05 using independent samples t-test between genders

^a^ Participant numbers vary due to the use of age-specific questionnaires; see Appendix A for details

Abbreviations: F, female; M, male

### Fatigue and pain

Fatigue was reported by 89.6% of the study population, with a mean fatigue VAS of 3.17 ± 2.63 (range 0–10). The CIS questionnaire was completed by 76 participants due to its age-related restriction (applicable for 11 years and older). Problematic fatigue (i.e. CIS total score > 76) was observed in 34.2% of these participants. Severe fatigue (i.e. score ≥ 35 on the CIS-Subjective Fatigue subscale) was present in 30.3% of participants. We found no significant differences between female and male participants with MO.

Pain was reported by 75.4% of participants, with a mean pain intensity score (VAS) of 2.41 ± 2.42 (range 0–10). The mean number of pain locations was 3.04 ± 3.09. We observed no significant gender differences in pain characteristics. Table 2 details fatigue and pain in children with MO and by gender.

**Table 2:** Dependent variables fatigue and pain

	*N* (Female; Male)	Total population Mean ± SD	Female Mean ± SD	Male Mean ± SD	*p*-value male vs. female
VAS Pain	134 (F:63 M:71)	2.41 ± 2.42	2.57 ± 2.52	2.27 ± 2.34	0.815
VAS Fatigue	134	3.17 ± 2.63	3.68 ± 2.88	2.72 ± 2.31	0.072
CIS-total	76 (F:38 M: 38)	66.32 ± 24.63	66.58 ± 26.98	66.05 ± 22.40	0.927
CIS-subjective fatigue	76	27.74 ± 13.60	29.21 ± 14.77	26.26 ± 12.33	0.348
CIS-severe fatigue^†^ (%)	76	23 (30.3%)	14 (36.8%)	9 (23.7%)	0.312

Abbreviations: F, female; M, male; VAS, Visual Analogue Scale; CIS, Checklist Individual Strength

^†^CIS-severe fatigue was defined as a score ≥ 35 on the subjective fatigue domain of the CIS questionnaire

Fatigue scores, compared with reference scores from healthy Dutch controls [20], are outlined in Table 3. Males with MO in our cohort reported significant higher fatigue scores compared with their healthy peers, both in the total CIS and in subjective fatigue subdomain (respectively, *p* < 0.001 and *p* = 0.017).

**Table 3:** Comparison of fatigue scores with healthy controls

	MO Mean ± SD	Healthy controls ^a^ Mean ± SD	*p*-value	95%CI
**CIS-total (*n*** **= 76)**	66.32 ± 24.63	55.07 ± 20.99	0.012*	2.64;20.38
Females (*n* = 38)	66.58 ± 26.98	59.06 ± 22.43	0.094	-1.35;16.39
Males (*n* = 38)	66.05 ± 22.40	50.98 ± 18.55	< 0.001*	7.71;22.44
**CIS-subjective fatigue (***n* **= 76)**	27.74 ± 13.60	24.44 ± 12.04	0.054	-0.09;9.63
Females	29.21 ± 14.77	27.54 ± 12.68	0.49	-3.19;6.53
Males	26.26 ± 12.33	21.26 ± 10.44	0.017*	0.95;9.06

Abbreviations: MO, Multiple Osteochondromas; CIS, Checklist Individual Strength

* *p* < 0.05 one sample t-test

^a^ Reference scores from ter Wolbeek et al. 2006 (The Netherlands) [20]

### Variables associated with fatigue and pain

Appendix B describes the first step of the two-step analysis, i.e. the univariable analyses of fatigue and pain using purposefully selected independent variables from the ICF model (Fig. 1).

Table 4 presents the multivariable regression models for fatigue, as quantified by VAS and CIS-total scores. The final VAS-Fatigue model explained 57.6% of the variance, with two variables remaining. Higher pain intensity and higher CBCL scores, indicative of behavioral and emotional problems, were both independently associated with increased fatigue scores as measured by VAS. Pain intensity accounted for 14.2% unique variance, while CBCL-total problems explained an even greater unique variance of 20%.

The fatigue model measured by CIS-total scores explained 52.4% of the variance, with three independent variables remaining in the final model. In this model, pain and CBCL score were again retained as independent variables, with pain intensity accounting for the largest unique variance (8.5%). The third variable in this fatigue model was CDI, which accounted for 3.6% of the unique variance. Higher CDI scores, indicating more severe depressive symptoms, were associated with increased fatigue levels.

**Table 4:** Summary of backward multiple linear regression analysis for fatigue (VAS & CIS-total) as dependent variable

Model 1: Fatigue – VAS	Unstandardized ß	ß	95% CI	Adj. *R*^2^	F	*p-*value	sr^2^	VIF
				0.576	51.96	< 0.001		
Pain – VAS	0.441	0.415	0.265–0.616			< 0.001	0.142	1.215
CBCL – total problems	0.086	0.493	0.057–0.115			< 0.001	0.200	1.215

**Model 2: Fatigue – CIS**								
				0.524	28.51	< 0.001		
Pain – VAS	3.252	0.889	1.480–5.025			< 0.001	0.085	1.215
CBCL – total problems	0.454	0.222	0.011–0.897			0.045	0.027	2.823
CDI	1.083	0.453	0.181–1.985			0.019	0.036	2.602

Abbreviations: VAS, Visual Analogue Scale; CIS, Checklist Individual Strength; CBCL, Child Behavior Checklist; CDI, Child Depression Inventory; R^2^ = adjusted explained variance, sr^2^ = squared semi-partial correlation, VIF = variance inflation factor

Table 5 presents the final multiple regression model for pain intensity (VAS) in MO. Two variables remained in the model, explaining 45.4% of the total variance in pain scores. Fatigue (VAS) was independently associated with pain (VAS), accounting for 9.2% of unique variance. For each one-point increase in VAS fatigue, VAS pain increased by 0.369 points. Higher functional disability (FDI) was also associated with greater pain intensity and explained 8.1% of unique variance.

**Table 5:** Summary of backward multiple linear regression analysis for pain (VAS) as dependent variable

Model Pain - VAS Independent variable	Unstandardized ß	ß	95% CI	Adj. *R*^2^	F	*p-*value	sr^2^	VIF
				0.454	32.21	< 0.001		
VAS fatigue	0.369	0.391	0.162–0.575			< 0.001	0.092	1.657
Functional Disability Inventory (FDI)	0.102	0.367	0.041–0.162			0.001	0.081	1.657

Abbreviations: VAS, Visual Analogue Scale; Adj. R2 = adjusted explained variance, sr2 = squared semi-partial correlation, VIF = variance inflation factor

## Discussion

This study demonstrates a high prevalence of fatigue and pain in the paediatric MO population, with especially male participants reporting higher fatigue scores compared to healthy gender matched reference values. To our knowledge, this is the first study to investigate the relationship between fatigue, pain, and other relevant variables in children and adolescents with MO. Consistent with findings in other chronic conditions, a significant association between fatigue and pain was observed in this population. Furthermore, our results highlight the importance of incorporating psychosocial assessment into the care of children with MO, as depressive symptoms were significantly positively associated with fatigue, and behavioural and emotional problems were linked to both higher levels of fatigue and pain.

### Fatigue and associated variables

Fatigue was highly prevalent in our cohort, with 89.6% of participants affected and about one third reporting problematic or severe fatigue. Males with MO also scored significantly higher on fatigue measures compared to healthy peers. To date, only two studies have examined fatigue in MO. One, conducted exclusively in adults, reported a similar prevalence of 90.4% [4]. The other included a mixed population of 11 children and 22 adults and found higher fatigue scores compared to healthy controls. Among adults, a prevalence of 85.7% was reported, while a specific prevalence rate for pediatric patients was not provided [5]. Owing to the small sample size, no formal analyses of associations were conducted in that study, but higher fatigue scores were observed in individuals with greater pain intensity and more pain locations. The adult study also identified associations between fatigue, pain intensity, number of pain locations, and anxiety and depressive symptoms, which are mostly consistent with our findings. In studies of other chronic pain conditions, fatigue has been identified as a significant mediator of the effects of pain intensity, sleep disturbance, and depressive symptoms on outcomes such as pain interference, mobility, and school functioning [13, 14, 31]. Notably, Yoon et al. also reported an association between depressive symptoms and fatigue in chronic pediatric pain disorders, which is consistent with our findings [14]. The literature describes a complex interplay between pain, fatigue, and psychosocial symptoms, which can significantly impact quality of life and impair school performance in affected children [14, 16, 32]. In MO, physical changes can be visible to peers and may increase the risk of bullying, which is associated with the development of mental health problems [33, 34]. Addressing pain in isolation is likely insufficient; instead, a comprehensive, multimodal treatment approach of fatigue and psychosocial factors should be considered in the management of children with MO.

### Pain and associated variables

Pain was reported in 75.4% of our pediatric MO population in the Netherlands. This aligns with prior studies, such as Boarini et al. (2024), who reported a prevalence of pain/discomfort in 78.8% in their mixed pediatric and adult population (*n* = 128) [6]. Goud et al. reported a pain prevalence of 63% in Dutch children with MO (*n* = 99) and 83% in adults. In their pediatric cohort, pain was associated with negative perceptions of disease and school-related problems, and no association was found with the number of surgical procedures. However, Boarini did find a negative relationship between pain and number of surgical procedures, which we could not replicate in our study. The relationship between surgical treatment and pain in MO remains a topic of debate with some authors even advocating for a more conservative approach [1, 2, 7]. Darilek et al. found pain in 84% of their mixed pediatric and adult cohort, with patients who had undergone surgery being 3.8 times more likely to report pain [2]. However, causality cannot be established due to the retrospective design, and it is plausible that patients requiring more surgeries may have had a more severe phenotype, leading to greater pain and functional limitation. Notably, Darilek et al. also reported that 55.1% of their cohort experienced generalized pain. This raises the question whether pain in MO should be conceptualized beyond local nociceptive mechanisms.

Recent research in pediatric chronic pain populations suggests that pain widespreadness, rather than primary pain location, is associated with worse pain-related outcomes such as pain catastrophizing, fatigue, anxiety, and depression [15]. It is plausible that similar mechanisms of nociplastic pain are relevant in MO. While surgical resection of symptomatic osteochondromas can relieve local compression-related/nociceptive pain, patients often experience pain at multiple sites; alleviating pain at one location may unmask or shift the burden to other symptomatic areas, underscoring the importance of careful surgical indication. Nevertheless, a broader pain model that incorporates nociceptive, neuropathic, and nociplastic processes may be warranted. Future research should focus on clarifying these complex mechanisms to inform more effective, multidisciplinary pain management strategies for patients with MO. These studies should also directly compare surgically and non-surgically treated patients with MO, ideally using a prospective longitudinal design with PROMs collected before and after surgery and at follow-up (1–2 years), explicitly accounting for surgical indication and assessing pain beyond the surgical site, including general pain burden and pain widespreadness.

We found no association between age and pain or fatigue in our pediatric population, while previous studies reported increased pain prevalence with advancing age [1, 2, 6, 35]. However, these studies included mixed pediatric and predominantly adult populations, which may explain the discrepancy with our findings. Moreover, some of our measurement instruments have age-specific versions (i.e. by parent or self-reported questionnaires with questions most applicable for different developmental stages). While these are specifically developed and validated for specific age groups, these different versions may hamper detection of age as potentially relevant variable.

Another ongoing debate concerns the role of gender in disease severity. We hypothesized that male gender would be associated with increased pain intensity and fatigue due to potentially more severe phenotypes and a higher incidence of EXT1 mutations, previously described in literature [8–12]. In this study we found no gender differences in pain intensity, which is in line with several other studies [2, 3, 6, 16]. Interestingly, fatigue scores were significantly higher in boys with MO compared to healthy boys, whereas no such difference was observed among girls. This pattern may suggest that the impact of MO on fatigue is more pronounced in boys, potentially due to differences in physical activity levels, adaptation strategies, or social expectations regarding the expression of fatigue. However, as genotype and detailed phenotype were not assessed in our study, future research is necessary to clarify the relationship between mutation type, disease severity, and clinical outcomes such as fatigue and pain.

In our cohort, higher CBCL scores, which indicate greater behavioral and emotional problems, were associated with increased fatigue and pain. This finding is consistent with other pediatric chronic conditions, where a strong association exists between chronic pain and emotional burden in youth and their families [36]. In these populations, pain is also associated with internalizing disorders such as anxiety and depression, which negatively affect overall health and pain experience [36, 37]. 

### Strengths and limitations

Several limitations should be considered when interpreting these results. First, the cross-sectional design precludes any conclusions regarding causality. Second, selection bias may be a concern, as recruitment through an expertise center and the national patient association could have led to an overrepresentation of symptomatic or more severely affected individuals. To mitigate this, we also included patients without scheduled orthopedic appointments by inviting them from the hospital registry. Efforts were made to capture a wide spectrum of disease severity by recruiting from multiple sources. Despite these constraints, this study represents the largest pediatric MO cohort to date investigating fatigue and related outcomes.

Recall bias, a common limitation in survey-based research, was minimized by focusing on participants’ current symptoms.

The rarity of MO also meant that a formal a-priori power calculation was not feasible, and thus subtle associations may have gone undetected. The actual obtained sample size (134 completed questionnaires) ensured that the analysis remained informative and enhanced methodological rigor and the interpretability of the findings. Additionally, the study did not include genotype and/or phenotype data, which could be informative for future research on symptom variability and quality of life. Phenotype data would inform on the degree of skeletal deformity and functional limitation. Given the considerable clinical heterogeneity of the MO population, these factors may importantly influence fatigue and pain outcomes.

## Conclusions

This study demonstrates that fatigue and pain are highly prevalent in children and adolescents with multiple osteochondromas. Fatigue scores of boys with MO were significantly higher than those of healthy peers, and both fatigue and pain were associated with behavioural and emotional problems.

While the relationship between pain and surgical interventions remains complex and unresolved, our data suggest that a narrow focus on surgical management alone may be insufficient. A multimodal approach addressing physical, psychosocial, and mental health domains could be more impactful. Future research should investigate the underlying mechanisms contributing to fatigue and pain in MO, including genetic and phenotypic factors, and explore targeted interventions to improve treatment for pediatric patients with MO.

## Supplementary Information

Below is the link to the electronic supplementary material.

Supplementary Material 1

Supplementary Material 2

## Data Availability

The data that support the findings of this study are available on request from the corresponding author. The data are not publicly available due to them containing information that could compromise research participant privacy.

## Declarations

### Patient involvement

The study was supported by the national patient association ‘HME-MO vereniging Nederland’. The association recruited patients through placing calls on their website and newsletters. The association also cooperated in the assessment of the burden of the study. Once the study has been published, we intend to inform participants of the results through the website of the national patient association and will sent details of the results in a study newsletter suitable for a non-specialist audience. Preliminary results were presented at the 2024 National MO Day, where they were discussed and well received by patients and their families.

## Abbreviations

Adj. R²: Adjusted explained variance
BMI: Body Mass Index
BPAQ: Baecke Physical Activity Questionnaire
CBCL: Child Behavior Checklist
CDI: Children’s Depression Inventory
CIS: Checklist Individual Strength
FDI: Functional Disability Inventory
FOPQ: Fear of Pain Questionnaire
ICF: International Classification of Functioning, Disability and Health
M: Male
F: Female
MO: Multiple Osteochondromas
PCS: Pain Catastrophizing Scale
sr²: Squared semi-partial correlation
VAS: Visual Analogue Scale
VIF: Variance Inflation Factor
