# Supplementary material for: Fatigue and pain in children with multiple osteochondromas: a cross-sectional study
Source: Orphanet J Rare Dis. 2026 Jun 29;21:324. doi: 10.1186/s13023-026-04476-2 (PMC13587480; doi:10.1186/s13023-026-04476-2)
Supplement: Supplementary file 1 [file 13023_2026_4476_MOESM1_ESM.docx]

**Appendix A.**

**OVERVIEW OF QUESTIONNAIRES USED, BASED ON AGE-SPECIFIC VALIDATION AND APPLICABILITY**

| **Questionnaire** | | **4-5 yrs.  (n=7)** | | **6-7 yrs.  (n=23)** | **8-10 yrs. (n=28)** | **11-15 yrs. (n=54)** | **16-18 yrs. (n=22)** |
| --- | --- | --- | --- | --- | --- | --- | --- |
| VAS – Pain intensity |  | |  | |  |  |  |
| Number of Pain Locations |  | |  | |  |  |  |
| VAS – Fatigue |  | |  | |  |  |  |
| CIS – Fatigue |  | |  | |  |  |  |
| FDI |  | |  | |  |  |  |
| BQ – Activity |  | |  | |  |  |  |
| PCS | PF | | PF | | CF | CF | CF |
| CBCL |  | | PF | | PF | CF | CF |
| CDI |  | |  | |  |  |  |
| FOPQ |  | |  | |  |  |  |

**Legend**:
**Green:** Validated and administered in this age group
**Red**: Not validated and thus not used in this age group

**Abbreviations**:
PF = Parent Form, CF = Child Form, VAS= Visual Analogue Scale, CIS= Checklist Individual Strength, FDI= Functional Disability Inventory, BQ; BAECKE activity questionnaire. PCS= Pain Catastrophizing Scale, CBCL= The Child Behavior Checklist, CDI= Children’s Depression Inventory, FOPQ= Fear of Pain Questionnaire.
