# Supplementary material for: Fatigue and pain in children with multiple osteochondromas: a cross-sectional study
Source: Orphanet J Rare Dis. 2026 Jun 29;21:324. doi: 10.1186/s13023-026-04476-2 (PMC13587480; doi:10.1186/s13023-026-04476-2)
Supplement: Supplementary file 2 [file 13023_2026_4476_MOESM2_ESM.docx]

# Appendix B. Univariable linear regression analysis (first step of 2-step model)

A two-step analysis was applied for the multiple liner regression models. In the first step, independent variables derived from the ICF model (Figure 1) were analyzed using univariable linear regression. Variables with p ≤ 0.25 were subsequently included in the second step, the results of which are presented in the main text. The tables below display the findings from the first step of the analysis.

**Table 7. Univariable linear regression analysis for pain – VAS**

|  | **Dependent variable – Pain Intensity – VAS** | | | |
| --- | --- | --- | --- | --- |
| **Independent variables** | **Standardized coefficients** | **Adjusted R^2^** | **95% CI** | **p- value** |
| Nr. pain location |  |  |  |  |
| 1-2 | -0.174 | 0.023 | -0.333 ; -0.004 | 0.045 |
| 3-4 | 0.100 | 0.002 | -0.071 ; 0.265 | 0.252 |
| ≥5 | 0.471 | 0.216 | 0.328 ; 0.593 | <0.001 |
| VAS – fatigue | 0.666 | 0.439 | 0.559 ; 0.751 | <0.001 |
| CIS – total score | 0.537 | 0.279 | 0.354 ; 0.680 | <0.001 |
| PCS | 0.291 | 0.078 | 0.128 ; 0.439 | <0.001 |
| FOPQ | 0.440 | 0.183 | 0.239 ; 0.606 | <0.001 |
| CBCL total problems | 0.395 | 0.149 | 0.237 ; 0.532 | <0.001 |
| CBCL Externalizing | 0.232 | 0.046 | 0.060 ; 0.390 | 0.009 |
| CBCL Internalizing | 0.400 | 0.153 | 0.243 ; 0.537 | <0.001 |
| FDI | 0.629 | 0.390 | 0.497 ; 0.733 | <0.001 |
| CDI | 0.340 | 0.107 | 0.158 ; 0.500 | <0.001 |
| Baecke – Physical Activity | 0.004 | -0.014 | -0.222;0.229 | 0.975 |
| Age | 0.271 | 0.067 | 0.107 ; 0.421 | 0.002 |
| Gender | 0.063 | -0.004 | -0.108 ; 0.230 | 0.470 |
| BMI | 0.174 | 0.023 | 0.004 ; 0.334 | 0.045 |
| Comorbidity | -0.021 | -0.007 | -0.190 ; 0.149 | 0.808 |
| Positive family history MO | -0.041 | -0.006 | -0.209 ; 0.130 | 0.639 |
| Nr. Surgical procedures |  |  |  |  |
| 1-2 | 0.065 | -0.003 | -0.106 ; 0.232 | 0.457 |
| 3-4 | 0.002 | -0.008 | -0.168 ; 0.171 | 0.984 |
| ≥5 | 0.236 | 0.049 | 0.070 ; 0.390 | 0.006 |
| Illness duration | 0.226 | 0.044 | 0.058; 0.381 | 0.009 |

**Table 8. Univariable linear regression analysis for fatigue – VAS**

|  | **Dependent variable – Fatigue – VAS** | | | |
| --- | --- | --- | --- | --- |
| **Independent variables** | **Standardized coefficients** | **Adjusted R^2^** | **95% CI** | **p- value** |
| VAS – pain | 0.666 | 0.439 | 0.559 ; 0.751 | <0.001 |
| Nr. pain location |  |  |  |  |
| 1-2 | -0.199 | 0.032 | -0.356 ; -0.030 | 0.021 |
| 3-4 | 0.116 | 0.006 | -0.054 ; 0.280 | 0.180 |
| ≥5 | 0.412 | 0.163 | 0.260; 0.543 | <0.001 |
| CIS – total score | 0.713 | 0.502 | 0.581 ; 0.808 | <0.001 |
| PCS | 0.257 | 0.059 | 0.091 ; 0.409 | 0.003 |
| FOPQ | 0.536 | 0.277 | 0.353 ; 0.679 | <0.001 |
| CBCL total problems | 0.533 | 0.278 | 0.395 ; 0.647 | <0.001 |
| CBCL Externalizing | 0.339 | 0.108 | 0.175 ; 0.484 | <0.001 |
| CBCL Internalizing | 0.575 | 0.325 | 0.446 ; 0.681 | <0.001 |
| FDI | 0.638 | 0.402 | 0.508 ; 0.740 | <0.001 |
| CDI | 0.577 | 0.326 | 0.432 ; 0.692 | <0.001 |
| Baecke – Physical Activity | -0.063 | -0.010 | -0.284;0.165 | 0.592 |
| Age | 0.327 | 0.100 | 0.167 ; 0.470 | <0.001 |
| Gender | 0.184 | 0.027 | 0.015 ; 0.343 | 0.033 |
| BMI | 0.182 | 0.026 | 0.013 ; 0.341 | 0.035 |
| Comorbidity | 0.149 | 0.015 | -0.022 ; 0.310 | 0.087 |
| Positive family history MO | -0.065 | -0.003 | -0.232 ; 0.106 | 0.458 |
| Nr. Surgical procedures |  |  |  |  |
| 1-2 | 0.157 | 0.017 | -0.013 ; 0.318 | 0.071 |
| 3-4 | -0.035 | -0.006 | -0.203 ; 0.136 | 0.689 |
| ≥5 | 0.218 | 0.040 | 0.050 ; 0.373 | 0.012 |
| Illness duration | 0.239 | 0.050 | 0.073 ; 0.393 | 0.005 |

**Table 9. Univariable linear regression analysis for pain – CIS**

|  | **Dependent variable – Fatigue – CIS** | | | |
| --- | --- | --- | --- | --- |
| **Independent variables** | **Standardized coefficients** | **Adjusted R^2^** | **95% CI** | **p- value** |
| VAS – pain | 0.537 | 0.279 | 0.354 ; 0.680 | <0.001 |
| Nr. pain location |  |  |  |  |
| 1-2 | -0.331 | 0.098 | -0.518 ; -0.114 | 0.003 |
| 3-4 | 0.100 | -0.003 | -0.128 ; 0.319 | 0.388 |
| ≥5 | 0.421 | 0.166 | 0.216 ; 0.591 | <0.001 |
| VAS – fatigue | 0.713 | 0.502 | 0.581 ; 0.808 | <0.001 |
| PCS | 0.380 | 0.133 | 0.169 ; 0.558 | <0.001 |
| FOPQ | 0.485 | 0.225 | 0.291 ; 0.640 | <0.001 |
| CBCL total problems | 0.650 | 0.414 | 0.497 ; 0.763 | <0.001 |
| CBCL Externalizing | 0.497 | 0.237 | 0.306 ; 0.650 | <0.001 |
| CBCL Internalizing | 0.638 | 0.399 | 0.481 ; 0.755 | <0.001 |
| FDI | 0.614 | 0.369 | 0.451 ; 0.737 | <0.001 |
| CDI | 0.627 | 0.385 | 0.468 ; 0.747 | <0.001 |
| Baecke – Physical Activity | -0.117 | 0.000 | -0.334 ; 0.111 | 0.312 |
| Age | 0.199 | 0.027 | -0.027 ; 0.406 | 0.084 |
| Gender | 0.011 | -0.013 | -0.215 ; 0.236 | 0.927 |
| BMI | 0.083 | -0.007 | -0.145 ; 0.303 | 0.476 |
| Comorbidity | 0.117 | 0.000 | -0.112 ; 0.333 | 0.315 |
| Positive family history MO | -0.180 | 0.019 | -0.390 ; 0.047 | 0.119 |
| Nr. Surgical procedures |  |  |  |  |
| 1-2 | 0.006 | -0.013 | -0.219 ; 0.231 | 0.957 |
| 3-4 | -0.103 | -0.003 | -0.321 ; 0.125 | 0.376 |
| ≥5 | 0.174 | 0.017 | -0.053 ; 0.385 | 0.132 |
| Illness duration | 0.050 | -0.011 | -0.177 ; 0.272 | 0.668 |

Abbreviations:

VAS – Visual Analogue Scale

CIS – Checklist Individual Strength

PCS – Pain Catastrophizing Scale

FOPQ – Fear of Pain Questionnaire

CBCL – Child Behavior Checklist

FDI – Functional Disability Inventory

CDI – Children's Depression Inventory

BMI – Body Mass Index
